# A mixed-methods descriptive study on the role of continuous quality improvement in rural surgical and obstetrical stability: Considering enablers, challenges and impact

**DOI:** 10.1371/journal.pone.0300977

**Published:** 2024-06-06

**Authors:** Jude Kornelsen, Audrey Cameron, Kathrin Stoll, Tom Skinner, Nancy Humber, Kim Williams, Sean Ebert

**Affiliations:** 1 Centre for Rural Health Research, Department of Family Practice, University of British Columbia, Vancouver, British Columbia, Canada; 2 Rural Coordination Centre of British Columbia, Vancouver, British Columbia, Canada; LSTM: Liverpool School of Tropical Medicine, UNITED KINGDOM

## Abstract

**Introduction:**

The Rural Surgical Obstetrical Networks (RSON) initiative in BC was developed to stabilize and grow low volume rural surgical and obstetrical services. One of the wrap-around supportive interventions was funding for Continuous Quality Improvement (CQI) initiatives, done through a local provider-driven lens. This paper reviews mixed-methods findings on providers’ experiences with CQI and the implications for service stability.

**Background:**

Small, rural hospitals face barriers in implementing quality improvement initiatives due primarily to lack of resource capacity and the need to prioritize clinical care when allocating limited health human resources. Given this, funding and resources for CQI were key enablers of the RSON initiative and seen as an essential part of a response to assuaging concerns of specialists at higher volume sites regarding quality in lower volume settings.

**Methods:**

Data were derived from two datasets: in-depth, qualitative interviews with rural health care providers and administrators over the course of the RSON initiative and through a survey administered at RSON sites in 2023.

**Findings:**

Qualitative findings revealed participants’ perceptions of the value of CQI (including developing expanded skillsets and improved team function and culture), enablers (the organizational infrastructure for CQI projects), challenges in implementation (complications in protecting/prioritizing CQI time and difficulty with staff engagement) and the importance of local leadership. Survey findings showed high ratings for elements of team function that relate directly to CQI (team process and relationships).

**Conclusion:**

Attention to effective mechanisms of CQI *through a rural lens* is essential to ensure that initiatives meet the contextual realities of low-volume sites. Instituting pathways for locally-driven quality improvement initiatives enhances team function at rural hospitals through creating opportunities for trust building and goal setting, improving communication and increasing individual and team-wide motivation to improve patient care.

## Introduction

The Rural Surgical and Obstetrical Networks (RSON) initiative in British Columbia, Canada, was proposed as a solution to enhance the health status of rural residents by stabilizing, supporting and enhancing British Columbia’s rural surgical and obstetrical programs [1]. This was done through funding for five mutually supporting program components, or “pillars”: increased scope and volume of rural surgical programs; clinical coaching and training opportunities; virtual care technologies to bridge distances between rural sites and larger, regional centres and to facilitate clinical coaching and backup support; a comprehensive evaluation of network implementation including patient outcomes and provider experiences, and a Continuous Quality Improvement (CQI) structure, to support team function and enhance local care. The RSON evaluation initially included seven rural communities across BC, though three more communities joined the program after the initial start date. This paper documents the process of the RSON-supported CQI framework from the perspective of rural care providers to better understand the enablers, challenges and values of CQI as part of an integrated suite of interventions to enhance rural procedural and obstetrical care.

Ensuring patient safety and providing quality care is the cornerstone of a learning health system where quality is defined as care that is safe, effective, patient-centered, timely, efficient and equitable [2]. This is reflected through the growing interest in and use of Donabedian’s model for quality of care, a scheme that considers structure, process, outcome and experience when defining and measuring quality and quality improvement in health care organizations [3]. Within this attention to quality care, growing research has emphasized the importance of *provider-driven* initiatives examining and responding to each of these domains of quality [4–6] to ensure relevance to local conditions. This is particularly salient in rural settings where local practice conditions may vary from larger centres where CQI models are built and implemented, and where additional resources may be needed (e.g., to travel and locum relief to participate in regional CQI events). Additional challenges to implementing CQI in rural settings include limited funding opportunities and heavy generalist provider workloads [7, 8].

Despite the well-documented need for care provider leadership in local quality initiatives, systemic challenges exist, such as the tension between time devoted to quality initiatives and time needed to fulfil clinical responsibilities, a tension which may disincentivize health care providers from taking on leadership roles [9]. Additionally, organizational and system-level influences may affect participation in CQI, namely poor compensation, lack of locally relevant CQI activities and structures and lack of time outside of clinical responsibilities [10]. A rural CQI needs assessment done in British Columbia found that half of participants had not engaged in any CQI activity in the last two years although most had a positive attitude towards the advantages of CQI [11]. This is despite research that demonstrates participant experience in quality improvement projects provides a direct line to the development of effective intra-team communication and trust, both key qualities of highly functional teams [12].

The RSON initiative set out to strategically address these issues by providing a pathway to enhance local participation in CQI programs. Although contingent in part on funding to allow for leadership and compensated time for the interdisciplinary teams’ participation, program supports also included a centralized framework to aid in site-level implementation, regional connections between the sites for the scale and spread of innovations, the creation of roles specifically to support CQI initiatives, and the development of instruments such as a Patient Reported Outcomes and Experience Survey to collect local data and inform potential CQI projects. The initial RSON initiative was active between November 2018 and May 2023. This allowed for iterative feedback and program adjustments at the participating sites. As part of the larger evaluation of RSON, which paralleled the RSON initiative, we recognize the importance of understanding and documenting the experience and insight of clinical and other rural partners to supporting CQI initiatives in rural settings. This paper reports these findings.

### Background

The Canadian Quality and Patient Safety Framework for Health Services identifies people-centred, safe, appropriate, accessible and integrated care as the overarching goals for quality improvement in health care [13, p.5]. Small, rural hospitals face barriers in implementing quality improvement initiatives [14] due primarily to lack of resource capacity, limited on-site quality infrastructures and the need to prioritize clinical care when allocating limited health human resources. Given this, a funding and resource stream for Continuous Quality Improvement was a key enabler of the RSON initiative and seen as an essential part of a response to assuaging concerns of specialist at higher volume sites regarding quality in lower volume settings. British Columbia has an established framework for quality improvement in health care through Health Quality BC which articulates seven dimensions of quality (five related to individual experience and two focusing on system performance) [15]. There is provincial funding, training and education for CQI supported by the provincial Physician Quality Improvement initiative [16], and separate funding for Nurse Practitioners [17] alongside other funding streams that may be inclusive of quality improvement initiatives [18]. There is a gap, however, in funding for team-based CQI, particularly funding that acknowledges and accommodates challenges to quality improvement initiatives in low-volume rural sites. Challenges include but are not limited to low procedural volume, the necessity for team-based care due to limited health human resources and the unique, contextually-determined circumstances of each community (where ‘one size does *not* fit all’). The RSON CQI funding filled this gap.

The objective of the funding stream or “pillar” itself was to improve local performance at an individual and team level, through promoting improvements to site processes, building the relationship between rural and referral sites and evaluating health outcomes based on validated, locally-identified quality measures [1]. The overarching approach to site-level quality improvement underscoring the funding was to appreciate local context-determined needs, identified by those working in patient care, provide a broad view of safety and quality levering on the local highly functioning teams, and provide local, funded leadership to implement projects [19, 20]. We did this through adopting a broad view of safety and quality inherent in Donabedian’s model for quality of care [3], to highlight rural team focus on ensuring safety of patients in rural and remote settings. In his health care quality assessment model, Donabedian identifies three components of quality: structure (the organizational context), process (activities and interactions that occur during the delivery of health care) and outcomes (consequences to the patient of health care interventions) [3]. In this context, the focus on *structure*, that is, the facilities, equipment and human resources delivering care, allows attention to the differential impact limited OR or recovery room space may have on care in a small facility. Likewise, assessing the currency and availability of local equipment determines the potential scope of local procedures and, consequently, ability to meet population needs. Although a focus on *process* may capture sequences of activities common across hospital settings, it also allows for focus on aspects of patient safety and quality that are unique to rural settings, such as efficient and effective transport to higher levels of care. This is salient in rural settings due to the agility low volume environments have in responding to patient assessments, as we explore below. The final component of the framework, *outcomes*, is rooted primarily in patient and family experience of receiving care and allows for more direct feedback on processes that influence quality, such as local involvement in decision-making.

RSON recognized the importance of a local quality champion and provided funding to support a half-time nurse lead to fulfil this role and ensure that outputs of the projects were woven into local and regional services, and provided an opportunity to identify continuing professional development and training needs. A further organizational priority was support for multidisciplinary teams [5, 21]. A proposed enabler of success was adopting CQI processes to a rural context to offset the usual urban orientation of quality projects [22, 23]. This includes attention to the impact of “low volume, inadequate reimbursement and investment, need for visionary leadership, staff training, professional staffing, inadequate information technology and a paucity of resources in general” [24, p.227]. Other research has noted the need for alternative methods of evaluating quality in rural settings, including a focus on “processes of care” [p.1378] as a metric for quality, as opposed to focusing on outcomes [22].

The approach to CQI was locally driven at each site. Although regular meetings were established, meeting intervals were not consistent between sites (some met weekly, some bi-weekly, and others met once per month). At these meetings, data collected at a site level was reviewed and strategic plans on addressing CQI findings were developed. There was provincial coordination for sharing results facilitated by the agency that administered the funding. This involved a quality network developed between sites; a structure for nurses to connect monthly to share project ideas, and opportunities for sites to engage in ongoing ‘CQI Dialogues’ that showcased successes. Findings were shared provincially through the RSON initiative’s annual meeting and annual provincial health care quality conference. Funding was available to compensate participants if they had to miss clinical time in order to engage in CQI.

Existing evaluations of rural CQI initiatives are often provider-led [5, 6] and benefit from on-the-ground leadership. Research suggests a focus on quality improves providers’ clinical knowledge and competence [19, 25] as well as patient outcomes [21, 26]. In some studies, the quality of care has been shown to improve through facilitating more timely access to the right care by the right person in the right place [6, 20, 27, 28]. Quality initiatives have also been shown to increase provider efficiency [7] and local team function [5, 19, 29] and increase adherence to best practice guidelines and clinical protocols [6, 29], in some instances resulting in reduced costs [6, 26].

Enablers of effective CQI in rural settings include optimal local-regional collaboration to allow for knowledge-sharing for patient coordination [30] and creating appropriate comparators for site-level data (i.e., other rural settings with similar contextual influences) [11]. This can be done through the use of networks to build regional “culture[s] of quality improvement” [8, Conclusion, para.2]. Most noted, however, is the value of a local champion [11] and the importance of initiatives being provider-driven [6], the latter facilitated by incentives for participation, financial support, administrative support and a supportive organizational culture [11].

## Methods

This study on health care providers’ and administrators’ experiences of and exposure to Continuous Quality Improvement falls under the Rural Surgical Obstetrical Network (RSON) initiative on the sustainability of rural surgical sites in British Columbia, for which funding and implementation of CQI was an interventional pillar. This was a mixed-methods descriptive study, with the effects of CQI funding assessed based on data derived from two triangulated datasets: in-depth, qualitative interviews and focus groups with rural health care providers and administrators over the course of the RSON initiative, and through a survey administered across RSON sites between February and March 2023. The approach to each is described below.

### Interview setting and participants

Participants were members of hospital teams, including providers, administrators and technicians, recruited by the study team with the assistance of community coordinators at each site, with four rounds of interviews performed between February 2019 and May 2022. The researchers had a longitudinal relationship with the research participants and introduced the wider objectives of the study prior to commencing. Participation was voluntary, and though not every participant was interviewed each year, no participants requested to drop out over the course of the study. The majority of participants were consistent over the course of the study. Each year, findings from the previous year were presented to participants, who were given the opportunity to provide comments on the data gathered. Study size was based on a convenience sample, including representatives from all RSON sites.

### Data collection

Data was collected through annual in-depth, open ended interviews between February 2019 to May 2022. Interviews were conducted in person or over Zoom videoconferencing software when necessary. The principal investigator (JK), experienced in qualitative interviewing and having worked to create rigorous evidence to support rural health decision making for the past two decades, led all interviews and was joined by a research assistant for note taking. The study received approval from the Behavioural Research Ethics Board of the University of British Columbia (BREB H18-01940). All participants provided recorded, verbal informed consent to participate before each interview, and each was educated on their right to withdraw their participation at any time. The University of British Columbia Behavioural Research Ethics Board approves of recorded verbal informed consent. All participants agreed to audio recording. Interview transcripts were transcribed by an external transcription service. Each transcript was anonymized and checked against the audio recording to ensure quality and accuracy of transcription. Transcripts were then moved into aggregate data for analysis. Interviews lasted between 30–60 minutes, with most reaching the 60-minute mark.

### Data analysis

Thematic analysis was used to interpret the qualitative data, following the steps outlined by Castleberry and Nolan [31], including compiling, disassembling, reassembling and interpreting the data. Researchers began by re-reading the transcripts and immersing themselves in the data. Using NVivo software, the data was then disassembled using a process of open-coding, wherein the data was broken down into discrete units and then inductively sorted into “meaningful groupings” [p.808], or codes. This simplified the dataset and organized it by interesting features. The codes then acted as identifiers across the dataset, allowing researcher to analyze all the data that was linked to that code [31].

The coded data was assembled into thematic hierarchies, with codes relating to participants’ experiences of CQI and “…put into context with each other to create themes” [31, p.809]. Using this framework, the study team generated overarching themes, which captured the patterns in experience of CQI between interviewees. This process was iterative, with researchers modifying codes and concepts as needed until the analysis yielded appropriate depth [32]. Although we were aware of data saturation, given the distributed nature of each site, when saturation occurred, we continued the interview process each year to ensure local nuances were captured.

#### Methodological rigor

To enhance the credibility of the analysis, researcher triangulation was used to improve the validity of the codebook. Independent coding was performed by two researchers who each developed a coding framework. Researchers then compared the two independent codebooks and found a high level of congruency between their codebooks. Researchers then collaborated to merge the coding framework and applied it to the entire dataset [33, 34]. A quality strategy of persistent observation was also employed, with researchers returning to the data throughout the process and adjusting the codebook as needed to accurately reflect the data [32]. Additionally, the authors responsible for coding the qualitative data engaged in ongoing processes of reflexive dialogue to ensure common understanding of the data and to mitigate bias.

### Survey design and participants

Quantitative survey data analysed within this study came from institutional data gathered by the Rural Coordination Centre of British Columbia. Ethics approval to do a secondary analysis of this institutional data was obtained from the Behavioural Research Ethics Board of the University of British Columbia (BREB H18-01940).

To help teams objectively measure team function and establish possible areas for improvement, data was gathered through an online version of the NHS Team Effectiveness Questionnaire (TEQ) tool. The TEQ was developed for the London Leadership Academy by the National Health Service and is made available for reuse through the Leadership Toolkit [35]. Designed to guide health care organizations, the tool quantifies team effectiveness across eight dimensions–purpose and goals, roles, team processes, team relationships, intergroup relations, problem solving, passion and commitment and skills and learning. There are seven questions per dimension, resulting in 56 questions total. Each question asks participants to select the number from 1 to 5 that best represents their view of how each question/statement describes their team–with 1 being ‘strongly disagree’ and 5 being ‘strongly agree’. The average score for each dimension is calculated, then added together to represent the overall effectiveness of a team out of a maximum cumulative score of 40.

Descriptive statistics were used to analyze and interpret survey data. Higher scores represent higher effectiveness, with 1 being the lowest, 5 being the highest, and a score of 3 being neutral. Based on this, a neutral cumulative score would be 24 out of 40 and higher scores would represent a more effective team [36, 37].

The original paper survey was put into an online format, with additional questions appended to the start of the survey to identify the hospital team the participant was on and their role. The survey was administered at seven of the RSON hospitals between February and March 2023, available through an email link and QR code posters in the hospital. Out of the 152 respondents, 87.5% completed the survey in its entirety (56/56 questions). All participants completed at least 95% of the survey. Given the high completion rate, all responses were included in the analysis.

## Results

### Survey findings

Recognizing that effective teamwork is foundational to other domains of quality improvement, RSON teams were invited to complete a 56-item team effectiveness survey that measured 8 domains of team function during the final two years of RSON [35]. Each domain includes 7 items with 5 response options: strongly agree (score of 5), agree (score of 4) neutral (score of 3), disagree (score of 2) and strongly disagree (score of 1). In addition to generating mean domain scores, a total score (average of 56 items) was also generated. Higher scores indicate better team effectiveness. A total of 152 responses were received from seven RSON sites. The number of submitted responses from each site ranged from 12 to 40. The internal consistency reliability (measured with Cronbach’s alpha) of the 56 items was very high (0.98; n = 132). Most surveys were submitted by nurses (n = 83), 39 surveys were submitted by physicians, 4 by midwives and 26 by other hospital staff (Administrative, Pharmacy, Housekeeping, Medical Device Reprocessing or Clerical team members). Though survey findings, in and of themselves, do not allow for direct correlation to be made with the RSON CQI work, the survey data provides nuance to the content of the qualitative interview data.

The average score for the 56 items was 4 out of 5, meaning that, on average, RSON team agreed with the items. The highest scoring domains were skills and learning (4.15), purpose & goals (4.11) and passion and commitment (4.12). The lowest scoring domain scores were 3.87 for team processes, 3.94 for intergroup relationships and 3.99 for roles (see Table 1). The standard deviations (SDs) can be seen as an indicator of agreement, with higher SDs showing less agreement and lower SDs more agreement. For example, the item ‘*Team members clearly understand their roles’* had a low SD indicating that people chose similar response options whereas the item ‘*As a team*, *we work to attract and retain top performers’* had a higher SD, indicating more diverse responses on this item. There were differences in the overall mean score depending on the role of respondents, with nurses scoring lower (on average 3.91) and physicians scoring higher (4.16).

**Table 1:** Mean item and mean domain scores (n = 152).

Question	N	Mean	SD
**TEAM RELATIONSHIPS**	**147**	**4.07**	**0.75**
Team members appreciate one another’s unique capabilities	150	4.22	0.88
Team members are effective listeners	152	4.01	0.88
Communication in our group is open and honest	151	3.92	0.97
Members of our team trust each other	151	4.03	0.96
Team members help one another deal with problems or resolve issues	152	4.16	0.79
We are able to work through differences of opinion without damaging relationships	151	3.89	0.92
Team members display high levels of cooperation and mutual support	152	4.22	0.84
**TEAM PROCESSES**	**149**	**3.87**	**0.70**
Team problem solving results in effective solutions	151	4.06	0.88
We address and resolve issues quickly	152	3.82	0.99
People on my team are rewarded for being team players	151	3.55	1.11
Group meetings are very productive	152	3.70	0.94
Our team has mechanisms in place to monitor its results	151	3.76	0.99
Our team works with a great deal of flexibility so that we can adapt to changing needs	152	4.22	0.73
When we choose consensus decision-making, we do it effectively	152	4.02	0.85
**SKILLS & LEARNING**	**148**	**4.15**	**0.61**
We have the skills we need to do our jobs effectively	152	4.22	0.69
We always ask ourselves, "How can we do better tomorrow what we did today?"	152	3.99	0.93
As a team, we are continually working to improve cycle time, customer responsiveness, or other key performance indicators	149	4.14	0.82
We view everything, even mistakes, as opportunities for learning and growth	152	4.16	0.81
We use various forms of training to keep our skills up-to-date	152	4.13	0.94
Team members embrace continuous improvement as a way of life	152	4.07	0.85
Team members work to ensure we are using best-practice methods	151	4.34	0.75
**ROLES**	**150**	**3.99**	**0.70**
Team members clearly understand their roles	152	4.23	0.75
When an individual’s role changes, an intentional effort is made to clarify it for everyone on the team	151	3.88	0.97
Team members understand one another’s roles	152	4.12	0.81
Everyone values what each member contributes to the team	152	4.09	0.92
Team members avoid duplication of effort and make sure they are clear about who is doing what	151	3.79	0.90
When team members’ roles change, specific plans are implemented to help them assume their new responsibilities	152	3.82	0.91
Overlapping or shared tasks and responsibilities do not create problems for team members	152	3.94	0.94
**PURPOSE & GOALS**	**150**	**4.11**	**0.68**
Our team has a meaningful, shared purpose	152	4.46	0.64
We are strongly committed to a shared mission	151	4.25	0.83
We focus on big-picture strategic issues as much as on day-to-day activities	152	3.86	0.99
We set and meet challenging goals	152	3.92	0.87
We consistently produce strong, measurable results	152	4.13	0.84
We make sure our work helps the organization achieve its goals	152	4.18	0.88
The mission and goals of my team are well aligned with the organization’s mission and goals	151	3.99	0.86
**PROBLEM SOLVING**	**148**	**3.86**	**0.68**
Team members take personal responsibility for the effectiveness of our team	151	4.05	0.89
Team members maintain a can-do approach when they encounter frustrating situations	151	4.15	0.74
Team members take initiative to resolve issues between themselves without involving the team leader	152	3.75	0.99
We spend very little time complaining about things we cannot control	151	3.27	0.99
Team members seek and give each other constructive feedback	151	3.74	0.99
Team members are sure about what is expected of them and take pride in a job well done	152	4.16	0.81
Team members consider how their actions will impact others when deciding what to do	152	3.93	0.94
**PASSION & COMMITMENT**	**151**	**4.12**	**0.70**
Working on our team inspires people to do their best	151	4.19	0.91
My team has a strong sense of accomplishment relative to our work	152	4.26	0.75
People are proud to be part of our team	152	4.32	0.84
Team members frequently go beyond what is required and do not hesitate to take initiative	152	4.17	0.87
As a team, we work to attract and retain top performers	152	3.78	1.13
Our team is excited about the contribution it is making to the organization’s viability	152	4.00	0.85
My team is proud of its accomplishments and optimistic about the future	152	4.08	0.95
**INTERGROUP RELATIONSHIPS**	**147**	**3.94**	**0.68**
We are able to resolve conflicts with other teams collaboratively	152	3.92	0.89
We seek to arrange our priorities to meet the needs of other work groups	152	4.01	0.91
We communicate effectively with other groups	151	3.89	0.88
Our team has established trusting and supportive relationships with other teams	151	3.91	0.90
We work toward integrating our plans with those of other work groups	150	3.85	0.81
Our collaborations with other teams are productive, worthwhile, and yield good results	151	3.98	0.80
The goals of our group support those of other groups	152	4.00	0.79
**TOTAL**	**132**	**4.02**	**0.63**

### Interview findings

A total of 169 participants from ten rural RSON communities were interviewed or attended focus groups over the course of the evaluation. Participants were professionals from a broad range of health disciplines, including Family Practice Anesthetists, Family Physicians, Family Physicians with Enhanced Obstetrical Surgical Skills, Registered Nurses, Midwives, medical reprocessing technicians, health service administrators, booking clerks and operating room managers. Through the course of the interviews and focus groups, several themes were generated regarding experiences with and observations of the CQI pillar. Broadly, these included the value of CQI, enablers, challenges in implementation and the essential role of CQI leadership. The themes and subthemes are listed in S1 Fig and are explicated below. Where applicable, we have also included relevant survey findings.

#### The value of CQI

*Improved skillsets and confidence*. Participants expressed that the value of local CQI initiatives included an expanded skillset and confidence as well as improved team function and culture, leading to measurable improvement in outcomes. Satisfaction in this area was reflected in responses to the quantitative survey as well, with participants reporting the highest ratings in the ‘Skills and Learning’ section (averaging to 4.15/5). Respondents strongly agreed with the statements that their teams work hard to use best-practice methods, and that they “embrace continuous improvement as a way of life.” Within the qualitative data, participants frequently noted that a focus on education supported the development of a broader range of skills and increased confidence in using those skills, particularly relevant for new graduates and those at low volume sites, who have less opportunities to reinforce their skillsets. Participants appreciated the repetitions afforded through practice simulations as well as CQI meetings, where teams review scenarios and significant clinical cases as a group. Several respondents discussed the impact low volume settings can have on provider confidence, particularly when practicing a broad, generalist skillset. As one respondent noted, “…you need to keep everybody sharp because you never know when it’s going to happen… and it’s devastating if you’re not ready for it.”

*Improved team function*. Most participants found that taking part in interdisciplinary quality improvement projects resulted in improved team function through the development of effective communication pathways and trust-building between providers. Participants also discussed increased motivation and desire to improve care *as* a team. Rather than accepting the status quo, or that something “is the way that it is”, participants reported that having CQI opportunities pushed teams to work together to improve their practice. “I feel like the bar has gone up, you know?” explained one participant, “I feel like we aim for higher, and we expect higher and if things aren’t higher, we try to work on what went wrong.”

Beyond the practical value of CQI in refreshing skills and improving patient care, participants also reported an increase in team cohesion, cultivated through expanded opportunities for collaboration. This often resulted in improved relationships between team members, increased openness to support one another and an enhanced learning environment. Results from the Team Effectiveness Survey showed that participants rated *team relationships* highly (domain score = 4.07); with average ratings ranging from 3.89 (“We are able to work through differences of opinion without damaging relationships”) to 4.22 (“Team members display high levels of cooperation and mutual support” and “Team members appreciate one another’s unique capabilities”). This suggests a positive perception of team relationships at the level of capability, honesty and trust.

The formalization of a safe learning environment provided opportunities for structured conversations clarifying roles and “who does what.” Relatedly, participants also noted an increase in comfortability with asking questions and trusting that the questions would be met with willingness to help rather than condescension or judgment. For many, this was a new experience for their health care delivery teams and reflected a shift in the local culture. Comfort with iterative learning was also reflected in the quantitative survey, where participants strongly agreed with the statement that their team views everything, even mistakes, as opportunities for learning and growth (4.16).

Participants also reported that CQI played a role in equalizing perceived hierarchy between different care providers. This occurred in part through CQI meetings, where all team members routinely came together to discuss quality projects. As one participant explained, “…everybody was in the same room, learning the same thing and eating the same food.” Additionally, some participants discussed the impact of running mock simulations as a part of their CQI, and expressed that simulations created opportunities for team members to find commonality, compare notes and experience and improve communication. As one participant noted, “I feel all of these activities have made us feel like a stronger team and we work really well together…. It’s been fantastic.”

*Measurable outcomes*. Many participants in this study felt that the consequences of local quality improvement–expanded skillsets and confidence and improved team culture and function–lead to measurable outcomes. This was described by some as a positive feedback loop, where the observable benefits of CQI initiatives noted above increased local ‘buy-in’, further improving care.

Many local teams started tracking patient feedback and outcome measures through local data collection initiatives (Patient Reported Outcomes and Experiences Surveys), using these reports to inform new CQI projects. Participants explained the importance of having more robust, site-specific feedback from patients, given some concern that singular instances of bad outcomes might skew the statistical value of data gathered at small volume RSON sites. This concern and the significance placed on patient feedback emphasized the importance of developing CQI frameworks appropriate to low volume settings and not based solely on traditional outcome measures.

#### Enablers of local CQI

Many participants reported that the RSON CQI funding gave their sites the organization and infrastructure they needed to facilitate quality improvement projects they had previously wanted to explore but did not have the means of initiating. Some projects were already underway and the protected CQI time and resources allowed teams to expand upon this existing work. For many, this also led to increased motivation and engagement. High ratings on the Team Effectiveness survey reflect this positive perspective, with participants strongly agreeing with the statement that their team has a meaningful, shared purpose (mean score of 4.46, the highest of the 56 scores). Qualitatively, participants shared that having a formalized CQI program allowed teams to protect time for integrating quality improvement opportunities into busy clinical schedules. As one participant explained, “…it’s essentially afforded us the time, especially as an OR team, to actually say right, let’s take on something that we feel we need to improve…”

*Support for project implementation*. Additionally, participants often discussed the value of protected time and funded leadership and coordinator positions to guide the development of a CQI program. Without this support and infrastructure, participants reported that CQI projects frequently fall to the wayside, and “just don’t get done.” The implementation of the CQI pillar was described as a “big shift…it felt very disorganized prior.” In contrast, the support meant that some hospital teams were able to “breathe life” into certain projects. Participants were excited to pursue further CQI initiatives, bolstered by the support they felt from leadership.

This support was also appreciated at sites where participants reported that quality improvement initiatives were already a part of the work culture and the expectations of team members. For example, one participant suggested that when work is being done informally, it is difficult to define how much time is spent on CQI, and exactly how effective it is. Most participants agreed that the funding helped formalize and add useful structure to quality improvement initiatives, as well as financial resources to compensate providers for their time.

*Local identification of needs*. Other participants discussed the importance of community-driven identification of relevant CQI projects which allowed sites to tailor quality initiatives and skillsets to their needs. The Patient Reported Outcomes and Experiences Survey (PROES) was developed and administered to patients undergoing procedural care at each site order to capture patient-identified areas of improvement and to organize patient feedback. Reflecting on results of the PROES feedback, most stakeholders identified it as an enabler of CQI. Participants also discussed the encouraging effects of positive comments from patients, sharing, “It has been an amazing team effort and morale booster for the OR staff, 100%. They love to see it. They love to read the comments…” Participants reported improved understandings of patient experiences of care following the implementation of the survey and the value of identifying where sites needed improvements according to patient perspectives. In some cases, the surveys helped “springboard” sites to higher levels on the Canadian Global Rating Scale for endoscopy, following the incorporation of patient feedback into practice. Generally, where surveys identified areas for improvement, sites were able to respond either through increased education or changes to process and protocol.

The additional emphasis on provider-driven identification of quality improvement needs meant there was room to prioritize “local gaps in services” and to develop and expand skillsets that are relevant and significant to each community. One participant highlighted this as important for rural sites given that quality processes are often developed in urban, centralized hospitals and then expected to be taken up in rural sites, even if the process is not a good fit for a rural context. They explained, “I love the fact that we can work on things that really matter to us.”

Team commitment to continuously improving care through identifying service gaps was also reflected in quantitative data. Participants rated their teams highly on ability to continuously work to improve cycle times, responsiveness, and other key performance indicators (4.14).

#### Challenges to implementing continuous quality improvement programs

Along with the benefits noted above, participants reported challenges that surrounded the implementation of quality initiatives including a lack of structure, complications in protecting/prioritizing CQI time, difficulty with staff engagement and skepticism from some participants surrounding the value of CQI as a funding priority in the context of unmet clinical needs.

*Lack of structure*. Although many participants identified local flexibility as essential to projects’ success at their sites, others felt the lack of concrete direction challenging. For some, this resulted in an observed steep learning curve, where sites had to learn through trial and error how and what type of projects to focus on. In other cases, teams reported taking on too many projects without the time and resources to complete them all. This led some to reflect retrospectively on the potential value of increased centralized guidance, at least at initial stages, although some participants noted that through the process, their site had successfully worked through the prioritization phase and were able to successfully complete their projects. As one participant said, “We have a better, clearer picture of what we’re doing now… but for a while it was a bit stressful not knowing if we’re doing the right things or going in the right direction.” Though lack of direction was identified as a challenge, participants also highlighted the capacity of their sites to still come together and start valuable conversations surrounding CQI planning.

For a minority, however, lack of direction contributed to skepticism and decreased participant motivation to engage in projects. In some cases, participants reported others’ perceptions of CQI as a grand idea at the proposal stage, but without the supports or back up to make the theoretical proposals real. Participants also discussed desire for assistance on the “meaning making” side of projects, finding there to be at times a lack of follow through on data gathered through CQI projects. “I think we get hung up on gathering data and then not necessarily doing a whole lot with it.” In these cases, structural supports for putting findings into practice or reaching the “end point” of a project were felt to be insufficient.

*Shortages in health human resources*. One of the most common points participants discussed was the challenge of protecting time to work on quality improvement in the face of significant staff shortages. While many participants identified protected CQI time as an enabler of quality improvement, others expressed that in reality, and in the face of extreme staff shortages, that time was often forsaken for patient care, with CQI nurses being redeployed to clinical care. Because RSON funds supported a half-time CQI lead in each community, usually a Registered Nurse, some questioned the feasibility and prudency of keeping staff from the front lines when the existing teams are “pressed to the boards.” Although other CQI team members were compensated for time spent on CQI activities, they were not released from clinical responsibilities so experienced struggles to engage in activities due to the constant pressure to support team members on the floor. Through this, participants identified an internal conflict between the value of thinking “future-forward” and improving quality of care, while also managing the immediate, day-to-day challenges faced as a result of short-staffing over extended/indefinite periods. This internal conflict was increased by participants’ discomfort and concern with being perceived by others as not contributing clinically to the team.

Participants did not always agree on the value of CQI overall and its level of priority when compared to direct patient care. One participant reported, “…even when I was a CQI nurse, it wasn’t protected time. It was not seen as valuable use of me to be sitting at a computer when people needed help.” Other participant voiced different experiences, with many expressing the “vital importance” of protected time for quality initiatives, especially given its role in maintaining skills. “I think it’s hugely important and I think that especially in rural sites that are lower volume you need to have [CQI] as an ongoing thing.” These participants maintained that the cost of sacrificing quality initiatives is that over time, this would cause skills to fall behind and compounds challenges in the flow of practice.

As one participant summarized, “…if you don’t keep it up, you lose it.” Nevertheless, most participants reported that when there were staff shortages that left nurses below baseline, time spent organizing CQI was the “first thing to go.”

*Staff engagement*. Some participants explained they were hesitant to participate in CQI due to the perception that it would be a significant addition to their already-overburdened workload. One participant expressed a lack of clarity surrounding whether or not CQI is a part of their role, or something in *addition* to their current workload. For this participant, buy-in was dependent on how much additional work would be required, given that they are not in a place where they have any extra time to take on more tasks. CQI was again identified by participants as one of the elements that falls to the wayside when staff are struggling to “keep heads above water.” Despite concerns of overloading and burning out providers, many participants described having “great participation” and well-attended CQI events.

Others reported seeing both people who were highly engaged and on board, as well as some who did not really “see the point.” This aligned with quantitative data, which showed participants gave slightly lower ratings in response to the statement, “Group meetings are very productive” (3.70). Data from interviews can provide some potential context, based on feedback from participants who reported challenges with engaging in interdisciplinary training, at times feeling their skills were irrelevant to the project at hand and subsequently finding it difficult to stay invested and involved.

In some cases, CQI was seen as something which can take a back seat when a site is clinically busy, further diminishing staff engagement. In response to this, one participant explained that they are looking for someone else who is willing to “champion” CQI initiatives, hoping that leadership initiative will increase staff buy-in and engagement. In their eyes, strong leadership, and having someone who understands the importance of the role and of the site-specific issues was immensely important, not only in tailoring initiatives to local needs, but also in encouraging other team members to engage in the process to enhance effectiveness. While some items in the ‘Team Process’ section of the Team Effectiveness survey scored in the lower range compared to the relatively high average scores across the survey, respondents highly rated their team’s ability to work with a “great deal of flexibility” to adapt to changing needs (4.22). Lower scores were found, for example for items such as ‘people on my team are rewarded for being team players’ (3.55) and the average score for ‘team process’ as a category was 3.87.

#### Value of a dedicated CQI role

Many participants agreed that the role of the CQI lead, usually an RN, was essential for implementing key quality improvement projects. Some participants viewed the CQI nursing role as one of the most significant contributions of the CQI funding. However, this position was challenged due to the health human resource shortages at many of the sites. Participants in this study reported on the effects of constant “redeployment” of nurses from CQI tasks to the floor, namely the disruption in project flow, limiting the effectiveness of the programs.

Participants also voiced appreciation for having a point-person to connect and collaborate with when they had a suggestion or concern relating to quality at their site. “I think everybody uses [CQI nurse] as a resource that way. And she’s always open to suggestions and happy to look into things.” Participants were able to funnel suggestions to one person who would then explore the feasibility of proposed projects, ensuring continuity of initiatives and timelines. Reflecting the sentiment of many participants, one noted, “I would love to have that [post-RSON]. For sure. One hundred percent. Worth every penny.” Another noted, “Without that [CQI nurse], we are–we’re done. Like even, it doesn’t matter how many great ideas we have and how many team people we have. Without that person to keep us all together, things don’t happen.”

In addition to the organizational role, many participants highlighted the benefit of having a liaison between the Health Authority and the hospital site, acting as a quality lead. This was reflected in concerns about what would happen following the loss of RSON funding for a discrete CQI nurse position at the end of the initiative. As one noted, “I’m worried about the fall down effect in 2023, …if [CQI nurse] disappears, and I go back to the CQI model that I had…I’m going to have nurses feeling even more isolated and more unsupported.”

*Hiring for the CQI role*. Although in most sites the CQI lead was filled by an RN, several respondents felt that the role shouldn’t be filled by a *nurse*, given the already limited pool of nurses available. Some suggested dividing the role such that non-clinical elements could be delegated to an administrator. Participants voiced a pragmatic desire to best utilize available human resources, prioritizing patient care and minimizing the syphoning off of nursing time from patient-oriented to administrative roles. Others, however, emphasized the value of CQI for long-term stability, suggesting that even if it meant pulling a nurse from patient care, having someone with a nursing skillset and scope of understanding is worth the clinical loss. For these participants, nurses, working full time in busy hospital environments, have the scope and perspective to identify areas for improvement better than an outsider, or someone with exclusively administrative experience. “Nurses working full-time hours can see a million ways they’d like it to get better…” Though clinical experience was valued for the position, at some sites, non-clinical CQI coordinators were brought on with great success. Participants found that with the right skillset, passion for quality and connection to the local team, non-clinical coordinators likewise proved highly effective at creating meaningful change at hospital-wide levels.

When discussing who should fill the role of CQI lead, participants identified various strengths they’d like to see in the candidate. Some participants emphasized being “team-led,” and found that the role of CQI nurse would be best filled by somebody familiar with team dynamics and rural hospital function. Others valued the quality side of the role, suggesting that the CQI candidate should be passionate about quality and have skills in oversight and coordination. Others suggested the need for a manager to oversee the CQI nurse, though most emphasized the leadership skills of the CQI candidate themselves.

Some discussed their *own* interest in the designated CQI role as well, highlighting its appeal as a nursing position. Interest stemmed from a desire to “take a crack” at improving hospital procedures. Coming from an insider role and understanding the strengths and weaknesses at a site, one participant explained, “I feel like this is some work that can be done. I know we’ve been struggling at this…I want to see if I can make this better.” This intrinsic desire to help improve places of work was echoed by other participants.

#### Outputs

The PROES survey was a springboard to an array of projects across the sites. Projects informed by these patient surveys included revisions to pre- and post-operation information packages at almost all sites, text-message distribution of post-operation handouts and pain surveys and streamlined referral and consultation processes such as virtual pre-operation consultations, to name a few. Other projects related to improved cultural safety and accessibility of services.

Additional site-level projects included trauma team activation sessions, bradycardia simulations and the integration of remote presence technologies (focusing on set-up and orientation). Under the umbrella of maternity care, value was placed on simulation training to compensate for limited exposure to potential clinical scenarios including, for example, simulation training for the delivery of twins, perimortem cesarean section and post-operative cesarean section hemorrhage. Beyond simulation, quality initiatives in maternity care included assembling a stillbirth kit and undertaking skills reviews (maternity RN skills). Several sites identified the desire to improve ‘decision to incision’ time for cesarean sections by identifying and tracking crucial time points for non-urgent, urgent and emergent deliveries.

Specific operative quality initiatives included projects geared towards improving team culture, retention and accountability, expediting OR turn-around time and supporting skills refresh through regional and provincial conferences. Sites that identified OR turnover time as a priority put in place OR trackers to enable targets and adapted OR schedules to best reflect local practice and increase efficiencies. This resulted in improved turn-around times that ultimately reduced surgical wait times. According to site level feedback, this improved efficiencies and, consequently, patient safety.

One RSON site identified the need for improved and culturally sensitive patient and family information around pediatric dental surgery with the goal of quelling family anxiety. This led to commissioning a professional video for families preparing for dental surgery, thereby improving patient and family experience and reducing the need for one-on-one patient education.

A final example of the myriad of site level projects included introducing a maternity nurse on-shift coaching project to allow experienced maternity nurses to mentor and support nurses new to practice or new to maternity care in order to reduce the sense of isolation many feel when they are on-shift without any RN backup. On-shift coaching enabled dedicated antenatal, intrapartum and postpartum education without requiring travel to other sites.

### Limitations

As with all qualitative studies, this research was limited by the voluntary nature of participation leading to the potential of those with strong positive or negative predispositions to the topic expressing their views to the exclusion of those with less pronounced views. However, due to longitudinal nature of the study across ten sites, we feel that our study design mitigated this as there were opportunities over several years for participation. Likewise, the inherent potential for the researchers’ personal beliefs and opinions influencing interpretation of study findings exists, but was mitigated in this instance through a team approach to data collection and analysis (as opposed to a single individual) and strategies such as the comparison of independently-developed codebooks and frequent member checking with participating community members to assess accuracy. The quantitative data was potentially limited by self-selection bias of participants, again capturing those with strong reflections on the topic which may not be representative of the larger cohort of eligible respondents. Further, as recruitment was done through email distribution across communities and passive poster recruitment, we do not have an accurate response rate, making assessment of wider representativeness challenging. However, we posit that other common limitations such as recall bias, response bias and incomplete data were mitigated by the timing of the survey (during the project wrap-up phase), the anonymized nature of the responses and low incidents of missing data (as described in the methods section).

## Discussion

Findings from this study, documenting the application of an integrated CQI program across ten rural sites in British Columbia, demonstrate the importance of structural supports and key indicators for a rural quality improvement program. This is consistent with international literature on the antecedents and importance of CQI in optimizing health service delivery. Newham et al. [38] noted the importance of not only focusing on what methods of quality improvement are most effective, but also “why, when and where they work most effectively” [38]. This evokes the importance of *understanding local context* as a precursor to successful CQI implementation and the attendant recognition of the importance of those familiar with local conditions in structuring mechanisms of CQI [39]. Likewise, barriers reported in existing literature, including resource constraints, access to local coordination for CQI initiatives and the tendency of clinical priorities to overshadow CQI work [38] were replicated through our findings.

Reciprocally, enablers of CQI revealed through this study, such as a positive and supportive organizational culture, strong interprofessional linkages and long-term, stable funding, were also identified in previous explorations of successful CQI initiatives [40]. Additionally, the importance of *team function*, or the characteristics that allow teams to achieve common goals, was a commonality across the literature reviewed. This is particularly relevant to rural settings where, due to limited health human resources, team participants likely know one another. When advantages of this natural association play out within a context of positive regard for all team members, increased feelings of psychological safety enhance group learning due to lack of vulnerability. Participants in this study identified this context as an important part of their CQI success [41]. Finally, many authors noted the importance of ‘co-production’ between health care providers and community members to establish effective measures to evaluate improved local services [39–41], a mandate that was key to the RSON initiative.

Although fundamental principles of CQI–identifying, analyzing and improving processes within an organization–are universal, there are specific considerations and challenges in a rural setting that need to be considered. Most saliently, this includes resource constraints in low volume rural settings, including health human resource shortages, limited access to virtual technology and inadequate infrastructure. This requires that quality initiatives create systems and structures to work with within constricted resources. Likewise, the geographic isolation of many rural communities leads to the prioritization of iterative quality approaches to address challenges related to transportation, telehealth solutions and visiting providers to ensure local needs are being adequately met. Despite the growing reliance on virtual technology, further entrenched through the COVID-19 pandemic, some rural settings still have limited access to broadband (and cellular service), which may demand alternative modes of data collection and analysis. A strategic advantage in rural settings includes the capacity to leverage the often tight-knit community ties to involve local perspectives on CQI initiatives and the attendant adaptability inherent in smaller health care settings that allows more flexibility to respond to local context and changing circumstances then many urban counterparts.

Given these conditions, Donabedian’s model for quality care is particularly well-suited for assessing and evaluating the quality of rural health care services, including identifying deficiencies in the structures of health care delivery which allows for precise and effective interventions, include the challenges posed by lack of access to local services [3]. Further, the focus on process allows attention to the distinct cultural norms and preferences unique to each community through their inclusion in the assessment of health care quality. Attention to outcomes shines a light on potential disparities experienced by rural residents when compared to their urban counterparts, providing useful data for both service improvement and system-level advocacy for resources to promote equity.

According to participants in this study, key enablers of local CQI included local identification of appropriate quality initiatives and measures, interdisciplinary approaches to initiatives that are inclusive of all team members and funding to support CQI positions and activities. Dedicated time for providers further facilitated interdisciplinary collaboration between maternity and surgical nurses, physicians and midwives and other team members to focus activities on improving patient safety and care. CQI activities, however, have limited utility without attendant accountabilities to *implement* findings at a site level to ensure system improvement in responsive PDSA (Plan Do Study Act) cycles. In the case of the RSON initiative, as noted above, characteristics of rural sites, including small provider teams, strong relationships across provider groups and direct contact with local administrators increase site adaptability, to the benefit of ongoing CQI projects.

The team function survey showed that RSON teams rated elements of team function that relate most directly to CQI (skills, learning and team relationships) highly. While it is unclear whether the team skills needed to successfully plan, integrate and implement CQI activities preceded RSON funding or improved with funding, the high scores across domains provide evidence of strong team function at RSON sites between February-March 2023. The relatively lower scores on the intergroup relationships domain coupled with the lower overall team function scores among nurses point to areas where improvements might be needed. High scores on the passion and commitment domain provide empirical evidence for a phenomenon that has been described anecdotally for decades, i.e. that the sustainability of rural services often hinges on the passion, commitment & tenacity of a small number of providers.

The mixed-methods approach underscoring this study allowed for internal triangulation of data and provides more validity to the findings. That is, there was synergy at a high level between the qualitative interview and survey data, with both explicating unique areas of focus. The qualitative findings provided insight into the context, perceptions and experiences of providers and administrators reflecting an understanding of the accrued value of CQI initiatives (including an expanded skillset and confidence and improved team function), the antecedents of a robust quality program (support for implementation “from the ground up”) and challenges experienced by those participating (lack of structure, limited resources, challenges with staff engagement due to onerous clinical responsibilities). Juxtaposing these data with survey findings, which focused more on quantifying characteristics of team relationships and processes, skills and learning and roles, for example, allowed a more holistic understanding of rural CQI and contextualization of the quantitative findings. This triangulated approach allowed for the corroboration of findings and increased the overall reliability and validity of the study, in addition to increasing its applicability to policy and decision-making through converging of insights into real-world experiences and broader patterns and trends.

Although direct evaluation of discrete projects has only been done in a limited way, qualitative feedback by participants regarding both the efficacy of projects undertaken at local sites and of the value of strategic CQI funding with enough flexibility to respond to local conditions suggest a positive relationship with local team function and service stability. Further, data from this study has shown the productive synergy between CQI initiatives and clinical coaching, as an integrated suite of interventions, and their productive contributions to improved team function at both site regional levels. These findings have implications for health care policy in rural jurisdictions similar to the ones in the study, both across Canada, as well as internationally.

## Conclusion

Although quality initiatives have been prioritized across all health care domains internationally, attention to effective mechanisms *through a rural lens* is essential to ensure that initiatives meet the contextual realities of low-volume sites. Instituting pathways for locally-driven quality improvement initiatives enhances interdisciplinary team function at rural hospitals through creating opportunities for trust building and goal setting, improving communication and increasing individual and team-wide motivation centered around improving patient care. In this study, this is reflected in the survey data, revealing high levels of team function in several of the domains explored. Foundational to ongoing success, and success across other rural sites, is base funding for local CQI leadership, release time and to backfill for others across disciplines. Beyond enabling coordination of activities, this funding is also a proxy for valuing quality improvement activities.

## Supporting information

S1 Fig Qualitative themes and subthemes. (TIF)

S1 File COREQ (COnsolidated criteria for REporting Qualitative research) checklist. (DOCX)

S2 File Quantitative survey dataset. (PDF)
