# Supplementary figures and images for: A mixed-methods descriptive study on the role of continuous quality improvement in rural surgical and obstetrical stability: Considering enablers, challenges and impact
Source: PLoS One. 2024 Jun 6;19(6):e0300977. doi: 10.1371/journal.pone.0300977 (PMC11156343; doi:10.1371/journal.pone.0300977)

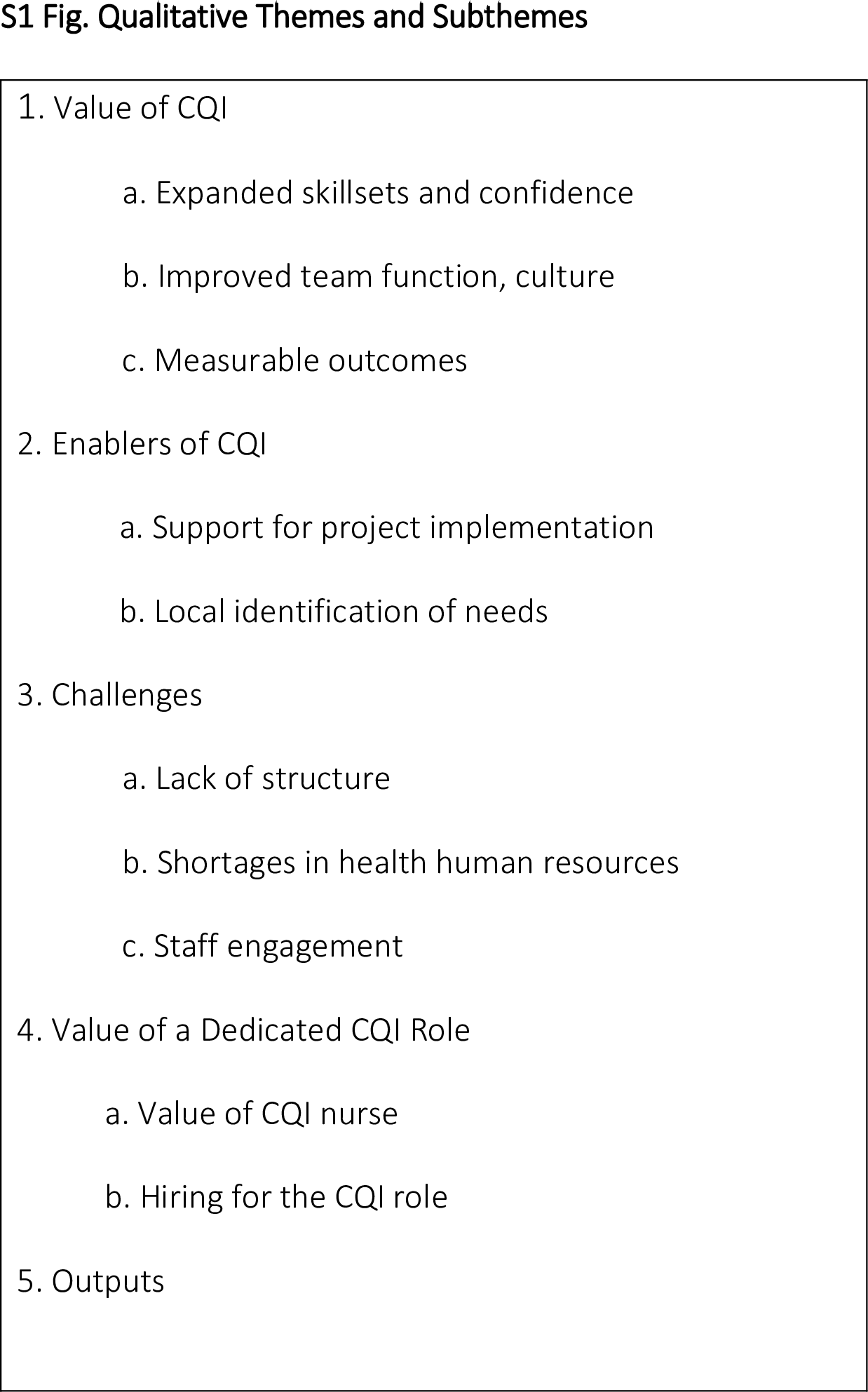

Supplement: S1 Fig — (TIF) [file pone.0300977.s001.tif]
