# Supplementary material for: A mixed-methods descriptive study on the role of continuous quality improvement in rural surgical and obstetrical stability: Considering enablers, challenges and impact
Source: PLoS One. 2024 Jun 6;19(6):e0300977. doi: 10.1371/journal.pone.0300977 (PMC11156343; doi:10.1371/journal.pone.0300977)
Supplement: S2 File — (PDF) [file pone.0300977.s003.pdf]

S3 File. Quantitative Survey Dataset

ID	Email	Our team has a meaningful, shared purpose	Team members clearly understand their roles	Team problem solving results in effective solutions	Team members appreciate one another's unique capabilities	We are able to resolve conflicts with other teams collaboratively	Team members take personal responsibility for the effectiveness of our team	Working on our team inspires people to do their best	We have the skills we need to do our jobs effectively	We are strongly committed to a shared mission	When an individual's role changes, an intentional effort is made to clarify it for everyone on the team	We address and resolve issues quickly
14	anonymous	5	4	3	4	3	4	3	4	4	4	3
15	anonymous	4	5	4	4	4	4	5	4	5	5	5
16	anonymous	5	5	3	4	4	4	4	3	5	3	3
17	anonymous	5	5	5	5	5	5	5	5	5	5	5
18	anonymous	5	5	4	3	4	5	5	4	4	4	3
19	anonymous	5	5	4	5	3	5	5	5	5	5	5
20	anonymous	3	3	2	3	3	2	2	4	3	2	2
21	anonymous	4	4	4	3	3	3	4	4	3	4	4
22	anonymous	4	4	2	3	3	2	4	4	3	3	2
23	anonymous	3	4	5	4	4	3	4	5	3	4	3
24	anonymous	4	5	4	5	4		4	4	4	4	2
25	anonymous	5	3	2	5	3	3	5	3	4	1	1
26	anonymous	4	4	2	3	2	2	2	4	2	2	2
27	anonymous	4	5	4	4	3	3	3	4	4	5	3
28	anonymous	4	4	4	4	4	4	4	4	4	3	4
29	anonymous	3	3	3	4	3	3	3	3	3	3	3
30	anonymous	4	5	4	4	4	5	5	4	5	4	4
31	anonymous	4	4	4	4	4	5	4	4	5	5	5
32	anonymous	4	5	5	5	4	5	5	4	4	4	4
33	anonymous	5	4	3	3	3	3	4	4	2	2	2
34	anonymous	4	3	4	4	3	4	4	4	4	4	3
35	anonymous	4	3	3	4	2	4	4	5	3	3	2
36	anonymous	4	3	3	2	3	2	2	4	3	1	2
37	anonymous	5	5	5	5	5	4	5	4	5	4	4
38	anonymous	5	5	5	5	4	4	5	5	4	4	4

[illegible]
